# STATc is a key regulator of the transcriptional response to hyperosmotic shock

**DOI:** 10.1186/1471-2164-8-123

**Published:** 2007-05-21

**Authors:** Jianbo Na, Budi Tunggal, Ludwig Eichinger

**Affiliations:** 1 Institute for Biochemistry I, Medical Faculty, University of Cologne, Joseph-Stelzmann-Strasse 52, 50931 Cologne, Germany

## Abstract

**Background:**

*Dictyostelium discoideum *is frequently subjected to environmental changes in its natural habitat, the forest soil. In order to survive, the organism had to develop effective mechanisms to sense and respond to such changes. When cells are faced with a hypertonic environment a complex response is triggered. It starts with signal sensing and transduction and leads to changes in cell shape, the cytoskeleton, transport processes, metabolism and gene expression. Certain aspects of the *Dictyostelium *osmotic stress response have been elucidated, however, no comprehensive picture was available up to now.

**Results:**

To better understand the *D. discoideum *response to hyperosmotic conditions, we performed gene expression profiling using DNA microarrays. The transcriptional profile of cells treated with 200 mM sorbitol during a 2-hour time course revealed a time-dependent induction or repression of 809 genes, more than 15% of the genes on the array, which peaked 45 to 60 minutes after the hyperosmotic shock. The differentially regulated genes were applied to cluster analysis and functional annotation using gene GO terms. Two main responses appear to be the down-regulation of the metabolic machinery and the up-regulation of the stress response system, including STATc. Further analysis of STATc revealed that it is a key regulator of the transcriptional response to hyperosmotic shock. Approximately 20% of the differentially regulated genes were dependent on the presence of STATc.

**Conclusion:**

At least two signalling pathways are activated in *Dictyostelium *cells subjected to hypertonicity. STATc is responsible for the transcriptional changes of one of them.

## Background

Virtually all cells, even individual cells in multi-cellular organisms, are subject to changes in the osmotic environment that are sometimes extremely rapid. In order to survive cells have to sense these changes and elicit an appropriate response that allows them to adapt. The response is complex and occurs in different phases. First, immediate cellular changes occur as a consequence of stress exposure, then defence processes are triggered and finally the cells adapt and resume proliferation [1].

In response to hypertonicity *D. discoideum *cells shrink immediately, they round up and rearrange their cytoskeleton, which appears to play a key role in the protection of the organism from high osmolarity. Actin is tyrosine phosphorylated and myosin II is phosphorylated on three threonine residues in the tail region [2-4]. Neither the signal transduction chain nor the responsible protein kinase for actin phosphorylation is known, however, there is evidence that the phosphotyrosine phosphatase PTP1 is somehow involved in the dephosphorylation reaction [2]. Myosin II phosphorylation appears to be triggered by the induction of soluble guanylate cyclase (sGC) which leads to a rise in cGMP levels and the activation of myosin II heavy chain kinase possibly via the cGMP binding protein GbpC [4-6]. Recent evidence suggests that the small GTPase Rap1 is involved in the cGMP response presumably by activating sGC [7]. Phosphorylated myosin II disassembles from myosin filaments followed by cellular relocalisation and reassembly. This apparently strengthens the cell cortex and is crucial for cell survival, as myosin II knock-out mutants and cells expressing mutant forms of myosin II, wherein the three threonine residues in the tail region were substituted by alanine, showed a dramatically reduced survival rate in high osmolarity [4]. Changes in the subcellular distribution of cell cortex proteins in response to sorbitol were also seen in two-dimensional gel electrophoresis with cytoskeletal and membrane fractions [8]. Furthermore, an increased sensitivity to hypertonicity was observed in double mutants of α-actinin and filamin, in hisactophilin mutants and in LimC, LimD and LimC/D mutants, supporting the importance of the actin cytoskeleton for the cellular resistance to an adverse osmotic environment [9-11].

A parallel pathway appears to be mediated by the hybrid histidine kinase DokA via a rise in intracellular cAMP levels. DokA minus cells showed a reduced viability on exposure to high osmolarity and artificial elevation of the intracellular cAMP concentration by 8-bromo-cAMP rescued this defect [12,13]. It is believed that activation of DokA by serine phosphorylation negatively regulates the RdeA:RegA two-component system which controls intracellular cAMP levels [13-15]. *In vitro *evidence suggests that DokA acts as a phosphatase for RdeA [13].

STAT proteins act as latent transcription factors and contain three highly conserved domains: a DNA binding site, an SH2 domain and a tyrosine phosphorylation site [16]. Analysis of *Dictyostelium *STATc knock-out cells showed that STATc regulates the speed of early development and the timing of terminal differentiation [17]. Developing *Dictyostelium *cells produce a chlorinated hexaphenone, DIF, which directs prestalk cell differentiation. In response to DIF STATc is activated by tyrosine phosphorylation, it dimerises, translocates to the nucleus and negatively regulates ecmA (a common marker used for prestalk cell differentiation) gene expression [17]. Recent work showed that STATc, which is present in growing cells and throughout development, is also activated by osmotic stress [18]. The link between STATc and the cAMP and cGMP signalling pathways is unclear. Although cGMP appears to be upstream of STATc, tyrosine phosphorylation of STATc was still observed in a *Dictyostelium *mutant wherein both known guanylate cyclases (GCA and sGC) were disrupted [18]. In this mutant, guanylate cyclase activity falls below detectable levels. Furthermore, DokA and protein kinase A (PKA) do not act upstream of STATc and cGMP accumulates after hyperosmotic stress in the dokA mutant [12,18].

In *Saccharomyces cerevisiae*, mammalian cells and plant cells diverse extracellular stimuli are transduced via MAPK cascades that function by activating a number of transcription factors thus regulating gene expression [19,20]. In *S. cerevisiae *the HOG signal transduction pathway, a MAPK pathway, plays a central role and controls via different transcription factors the expression of more than 150 genes [1,21]. Yeast cells subjected to hyperosmotic conditions adapt by synthesizing the compatible osmolyte glycerol. In addition, they respond by a whole range of physiological changes. The cytoskeleton is reorganised, ion homeostasis is changed, metabolic processes are adapted, the cell cycle is stopped and massive changes in gene expression are induced [1,22,23]. Parallel MAPK-cascades are also involved in the osmostress-induced signal transduction in mammalian cells [24]. In addition, mammalian cells activate specific JAK-STAT (Janus kinase-STAT) signaling pathways in response to osmotic or oxidative stress. Activation by osmotic shock is thought to be triggered by cell shrinkage and presumably acts also via a MAPK cascade [25-27]. In contrast to yeast, the responsible osmosensors in *Dictyostelium *and mammalian cells are unknown.

We performed gene expression profiling using DNA-microarrays to better understand the *Dictyostelium *response to hyperosmotic conditions. Treatment of cells with high osmolarity over a two hours time course resulted in the transcriptional regulation of more than 15% of the genes on the array. Two main responses appear to be the down-regulation of the metabolic machinery and the up-regulation of the stress response system, including STATc. We also find an enrichment of differentially regulated genes involved in fruiting body formation, which is consistent with the notion that the cellular processes that protect amoebae from a hypertonic environment have been adapted for regulatory developmental processes. Our results support the existence of at least two signal transduction pathways that are activated in *Dictyostelium *cells subjected to hypertonicity. The differential regulation of target genes in one of these pathways depends on the presence of STATc.

## Results

### High osmolarity triggers a variety of responses in *Dictyostelium *cells

**Figure 1:**
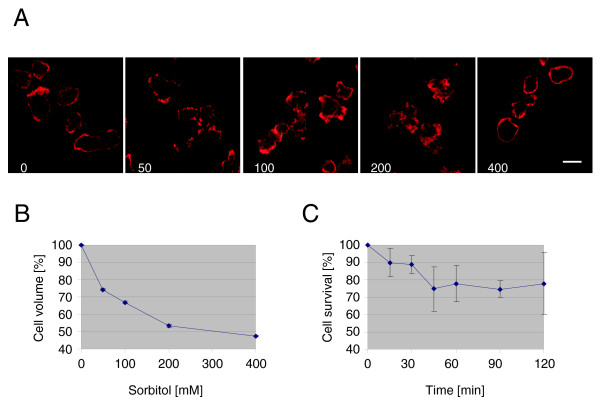
**High osmolarity triggers a variety of responses in *Dictyostelium *cells**. (A) Redistribution of the F-actin cytoskeleton in response to hyperosmotic shock. After starvation for 4 hours, untreated cells and cells treated for 5 min with 50, 100, 200 and 400 mM sorbitol were fixed with ice cold methanol, and then stained with a monoclonal antibody specific for actin, followed by the incubation with anti-mouse IgG antibody conjugated with Cy5. Size bar is 10 μm. (B) Decrease of cell volume in response to hyperosmotic condition. *Dictyostelium *cells were treated with increasing sorbitol concentrations for 5 minutes. Cell volume was measured with a microcapillary. Values represent the mean of three independent experiments ± standard deviation (SD). (C) Cell survival in response to hyperosmotic shock. Cell survival was measured by plating out treated or untreated cells on *Klebsiella aerogenes *lawns and counting the plaques after 2 days of incubation at 21°C. Values represent the mean of three independent experiments ± SD.

We treated *Dictyostelium *cells with increasing concentrations of sorbitol and analysed their cellular response with different methods. Immunofluorescence microscopy studies of starved *Dictyostelium *cells with a monoclonal antibody directed against actin confirmed the redistribution of the F-actin cytoskeleton in response to hyperosmotic conditions [4,28]. While untreated cells showed a polarized actin distribution, F-actin was redistributed in treated cells and appeared to form a continuous cortex at 400 mM sorbitol, which is thought to play a pivotal role in protecting the cells against the hyperosmotic environment. The analysis also showed that upon treatment the cells changed their shape and also decreased in size (Fig. 1A). We quantified the decrease in cell volume and found that it is strictly dependent on sorbitol concentration. At 50 mM sorbitol the cell volume decreased to 73 % and at 400 mM to 48 % of untreated cells. Extracellular concentrations of 100 and 200 mM sorbitol resulted in intermediate values (Fig. 1B). The decrease in cell size is due to loss of water resulting in a fast increase of the intracellular osmolarity of the treated cells until their osmolarity matches the surrounding medium [29]. In addition, we found that the *Dictyostelium *cells were able to adapt to 50 and 100 mM sorbitol and resumed the original cell size about one hour after treatment (data not shown). Next we checked cell survival by treatment with 200 mM sorbitol for different times. After 15 and 30 minutes about 10 % and after 45 minutes about 25 % of the cells had died and the latter value remained constant throughout the time course (Fig. 1C). Furthermore, Northern blot analysis showed that several genes encoding cytoskeletal proteins were differentially regulated upon treatment of cells with 200 mM sorbitol (data not shown).

These results exemplify the complex response of *Dictyostelium *cells to hyperosmotic conditions. To better understand this response we treated the cells with 200 mM sorbitol and analysed their global transcriptional response by using DNA microarrays.

### Hyperosmotic shock of *Dictyostelium *cells results in dramatic transcriptional changes

**Figure 2:**
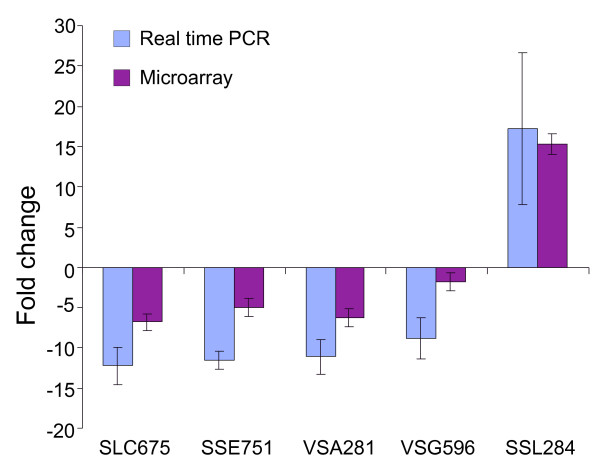
**Validation of microarray results by real time PCR**. Five ESTs were analysed by real time PCR and compared with the microarray results. The data are expressed as means of fold change ± SD of three independent experiments. The corresponding DDB IDs from left to right as follows: DDB0188166, DDB0235172, DDB0188166, DDB0190245, DDB0185120.

We employed cDNA microarrays to first analyse the transcriptional changes one hour after treatment with 200 mM sorbitol. Without additional threshold SAM (significance analysis of microarrays) reported 2007 genes as differentially expressed, of which 873 were up-regulated and 1134 down-regulated. Significantly more genes were down-regulated than up-regulated which suggests that *Dictyostelium *down-regulates many cellular processes in order to survive. We randomly selected five of these genes to validate the microarray results by real time PCR. The differential expression was confirmed for all five genes (Fig. 2). However, the absolute values for the fold change that were obtained with real time PCR were in all cases higher than those obtained with the microarray. The phenomenon that microarrays often provide compressed measurements in comparison to Northern blots or real time PCR is well known, however, its exact cause is not clear.

Next we analysed the transcriptional response in a time course experiment. The time points 15, 30, 45, 60, 90 and 120 minutes post treatment were chosen because of the microarray result described above and because of Northern blots where clear differential expression for several genes was already apparent 30 minutes after subjecting the cells to hyperosmotic conditions (data not shown). For analysis we hybridised six microarrays and thus obtained up to twelve measurements for each probe. As a consequence SAM reported many genes that were only slightly up- or down-regulated. Since very low folds of change cannot be confirmed with alternative methods and the biological impact of such small changes is unclear, an additional threshold for the fold change of 1.5 was used for the analysis of all experiments. To assess the likelihood of falsely reported differentially regulated genes we also compared the transcriptional profile of untreated cells against one another at t_0_. Under these conditions, only one gene was reported by SAM without additional threshold (data not shown) and no gene at a threshold of 1.5 (Table 1). This result confirmed the reliability of our analysis pipeline for the detection of differentially regulated genes. Differentially expressed genes were identified throughout the rest of the time course, but their number varied largely. 15 minutes after treatment and at a threshold of 1.5 only 38 genes were identified and, surprisingly, 35 of these were up-regulated. At 30, 45, 60, 90 and 120 minutes 485, 588, 583, 323 and 211 genes were found, respectively, and for all of these timepoints significantly more genes were down-regulated than up-regulated (Table 1). The largest number of genes was identified 45 and 60 minutes after treatment, when nearly 600 genes were differentially expressed. This corresponds to more than 10% of all spotted probes and indicates that the major transcriptional changes in *D. discoideum *occur between 30 and 60 minutes after treatment with sorbitol.

**Figure 3:**
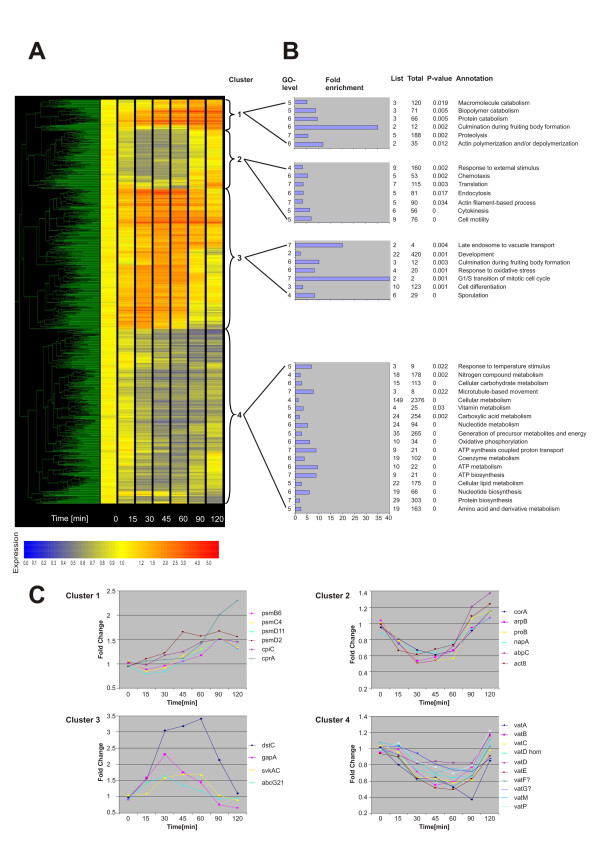
**Cluster analysis and functional annotation of the differentially regulated genes of the time course of sorbitol treatment**. (A) A non-redundant set of 809 genes that were up- or down-regulated more than 1.5 fold in the time course of sorbitol treatment was clustered with GeneSpring 7.2. Four major clusters (1–4) can be distinguished. The dendrogram is displayed on the left. The differentially regulated genes are depicted as coloured lines and the time of treatment in minutes is shown at the bottom. The colour represents the fold induction (red) or repression (blue) as shown in the colour scale below the figure. Non-regulated genes are displayed in yellow. (B) A selection of the GO biological process terms that were enriched in each of the clusters is presented. GO tree levels are shown on the left. Bar lengths represent the fold enrichment (scale x-axis). The table indicates the number of genes with a particular annotation in the cluster (List), on the entire array (Total), the significance for enrichment (P-value) and the annotation. (C) Expression profiles of selected genes from each cluster. The following abbreviations for differentially regulated genes are used. Cluster 1: *rto*A, *Ratio*A; *psm*B6, 20S proteasome subunit beta-6; *psm*C4, 26S proteasome subunit ATPase 4; *psmD*11, 26S proteasome non-ATPase regulatory subunit 11; *psmD*2, 26S proteasome regulatory subunit 2; *cpi*C, Cysteine Protease Inhibitor; *cpr*A, cysteine proteinase 1. Cluster 2: *cor*A, coronin; *arp*B, actin related protein 2; *pro*B, profilin II; *nap*A, NCK-Associated Protein; *abp*C, actin binding protein; act8, actin8. Cluster 3: *dst*C, *Dictyostelium *STATc; *gap*A, RasGTPase-activating protein; *svk*A, Severin kinase; *abc*G21, ABC transporter G family protein. Cluster 4: *vat*, vacuolar H+-ATPase subunit.

The expression profiles of the differentially expressed genes were used for a cluster analysis to identify groups of similarly regulated genes. The analysis was performed with GeneSpring 7.2 with a non-redundant set of 809 genes that was created from the 908 regulated genes of the time course (Additional file 1). Four major clusters of genes could be identified (Fig. 3A). The first cluster mainly contained genes that increased in expression throughout the time course with maximal expression at later timepoints, the second one was comprised of genes that were down-regulated between 15 and 60 minutes and then changed to a neutral level or were even slightly up-regulated, the third one was characterized by genes up-regulated mainly between 30 and 60 minutes and the fourth one by genes down-regulated throughout the time course.

### GO annotation shows the upregulation of stress response genes and downregulation of metabolism

A common challenge faced by researchers is to translate lists of differentially regulated genes into a better understanding of the underlying biological phenomena. This can be accomplished by the generation of a functional profile that is able to provide insight into the cellular mechanisms relevant in the given condition. However, several issues need to be considered when interpreting transcriptional changes in terms of physical adaptations. First, there is not always a direct connection between a change in mRNA content and a meaningful change of its protein product. Second, the proposed biochemical functions of many gene products are only based on homology to characterized proteins and might not be correct. And third, the consequences of slight changes in protein content to biological processes is seldom known with any confidence since the networks have yet to be defined. Therefore, the outcome of such analyses should be taken with care.

The GO [30] project is an effort to produce a system for annotating gene products that can be applied across all organisms. GO is divided into three categories describing biological processes, molecular functions and cellular components [31]. GO term enrichment was analysed with GOAT [32]. Enriched biological process GO terms of the four main clusters are shown in Fig. 3B. Only a selection of those GO terms that had a p-value < 0.05 are listed. The full list of all enriched biological process, molecular function and cellular component GO terms is available as supplementary information (Additional file 2). We first analysed clusters 1 and 3 which comprise up-regulated genes. GOAT analysis showed on the biological process level an enrichment of genes involved in actin polymerization and/or depolymerization, macromolecule catabolism and proteolysis for cluster 1. Interestingly, an enrichment of genes involved in culmination during fruiting body formation was also reported. On the cellular component level the proteasome complex was enriched (Additional file 2). Fig. 3C, cluster 1, depicts the expression profiles of six genes encoding proteases or proteasome subunits. These were first up-regulated 30 to 45 minutes after treatment and for most of them the expression further increased at later time points. The GOAT analysis for cluster 3 revealed on the biological process level an enrichment of genes involved in the response to oxidative stress, in late endosome to vacuole transport, in the G1/S transition of the mitotic cycle and in development, in particular culmination during fruiting body formation and sporulation. In addition, the GO molecular function terms showed for this cluster an enrichment of genes encoding transporters, transcriptional repressors, Ras GTPase activators and inhibitors, Ser/Thr protein kinases, Rho GTPase binding proteins and cytoskeletal proteins (Additional file 2). The expression profiles of genes assigned to the biological process category "response to oxidative stress", STATc (*dst*C), RasGAP (*gap*A), severin kinase (*svk*A) and an ABC transporter (*abc*G21) are depicted in Fig. 3C (cluster3). We will consider *Dictyostelium *STATc in more detail below.

As expected, the lists of enriched GO terms for the mainly down-regulated genes (clusters 2 and 4) differed considerably with the notable exception of genes encoding cytoskeletal and developmental proteins. Cluster 2 showed on the biological process level an enrichment of gene products involved in the response to an external stimulus, in translation and in cellular functions that require cytoskeletal proteins like endocytosis, chemotaxis and cytokinesis (Fig. 3B). The cellular component category revealed an enrichment of the cortical actin cytoskeleton and this was also reflected in the GO molecular functions where, among others, structural constituents of the cytoskeleton were reported (Additional file 2). The expression profiles of some genes encoding cytoskeletal proteins are shown in Fig. 3C (cluster 2). Cluster 4 is characterized by down-regulated genes, which either remained repressed throughout the time course or returned to normal levels at the end of the two hours treatment. On the biological process level genes whose products are involved in all aspects of metabolism were enriched indicating that the cells reduced their metabolic activities upon exposure to high osmolarity (Fig. 3B). Interestingly, all genes encoding the different subunits of the vacuolar ATPase were regulated in a very similar manner (Fig. 3C, cluster 4).

### The early transcriptional response to hyperosmotic stress

The early transcriptional response of the cells to hyperosmotic stress is particularly interesting because these genes are apparently primary targets of the signaling cascade(s) that redirect(s) the transcriptional program of the cells. Furthermore, individual components of the signaling cascade(s) might be differentially regulated and it is also feasible that the responsible transcription factor(s) autoregulate(s) its/their own expression via a positive or negative feed back loop. Therefore, we manually annotated and analysed the early differentially regulated genes of the time course experiment (Table 2). The analysis revealed several interesting up-regulated genes encoding STATc (see below), FcpA (a putative C-terminal domain phosphatase that could play a regulatory role in the response to hyperosmolarity) and RasGapA (an IQGAP-related protein involved in the completion of cytokinesis). In addition, genes for several transporters, two Cyclin_N domain containing proteins, RabR and the eukaryotic translation initiation factor 4E, which has a significant function in the initiation of eukaryotic protein synthesis, were reported. Interestingly, we also found a number of genes 15 minutes after treatment and at later time points that had been shown to be regulated by the MADS box transcription factor SrfA [33]. Therefore, we tested the role of SrfA in the *Dictyostelium *response to hyperosmotic conditions by comparing the transcriptional profiles of sorbitol-treated AX2 and *srf*A^- ^cells. No major differences in the transcriptional profiles were found, therefore, SrfA appears not to be involved in the transcriptional response to hypertonicity (data not shown).

### STATC is a key regulator of the transcriptional response to hyperosmotic stress

**Figure 4:**
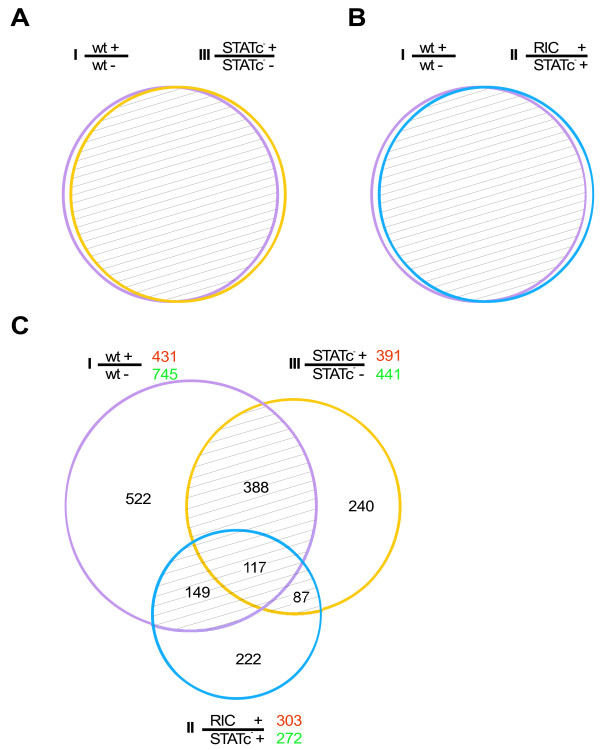
**STATC is an important regulator of the transcriptional response to hyperosmotic stress**. (A) Expected overlap of differentially regulated genes in experiments I and III if STATc is not involved in the transcriptional regulation. (B) Expected overlap in experiments I and II if STATc is the only transcriptional regulator in response to hypertonicity. (C) Venn diagram of the observed differentially regulated genes from the three comparisons: wt cells treated versus untreated (I), RIC cells treated versus STATc^- ^treated (II) and STATc^- ^treated versus untreated (III). The numbers of up- and down-regulated genes of the single experiments are printed in red and green, respectively. Genes shared between 2 or 3 comparisons (shaded region) were applied to further analysis.

A very interesting member of the early differentially regulated genes was STATc (Table 2 and Fig. 3, cluster 3). It had already been shown that STATc is activated in cells subjected to different types of stress [18] and we reasoned that STATc might be a transcriptional regulator in the *Dictyostelium *response to hyperosmotic conditions. To test this hypothesis we performed microarray experiments with either treated or untreated AX2 wt cells (wt; experiment I), the STATc knock-out mutant (STATc^-^, experiment III) and a mutant with an isogenic background where the STATc knock-out construct was randomly integrated into the genome (RIC, experiment II) (Table 3). Cells were treated for one hour with 200 mM sorbitol and the lists of differentially regulated genes were compared between the different experiments. There are several possible outcomes: i) If STATc is not involved in the osmostress response we would expect a complete overlap of the differentially regulated genes in experiments I and III (Fig. 4A). ii) If STATc is the only transcriptional regulator that directly gets activated in sorbitol-treated *Dictyostelium *cells we would expect no differential regulation in experiment III and a complete overlap of the gene lists in experiments I and II (Fig. 4B). iii) If STATc is only partially responsible for the transcriptional response we would expect a more complicated output. Of the three possibilities we found the latter to be the case. A set of 117 genes was identified, that was common to all three comparisons and sets of 388, 149 and 87 genes, respectively, that were common to two comparisons (Fig. 4C). The 149 differentially regulated genes that were common between experiments I and II appear to be solely regulated by activated STATc. In contrast STATc is only partially responsible for the regulation of the 117 and 87 differentially regulated genes, respectively. Interestingly, 82 of the 87 genes common between experiments II and III were oppositely regulated in these experiments resulting in a balanced output in experiment I (data not shown). We conclude that STATc is a major but not the only transcriptional regulator in the *Dictyostelium *response to hyperosmotic conditions. If we assume two parallel signaling pathways that are activated upon hyperosmotic stress and in addition a STATc pathway independent of osmostress we need to consider three factors that influence the transcriptional output of every gene in our comparisons: i) regulation by the osmostress pathway 1 (OP1), ii) regulation by the osmostress-induced STATc pathway (OSP) and iii) regulation by the STATc pathway (SP) irrespective of osmostress. The transcription of a given gene is either independent of these factors (majority of genes) or activated or repressed. Since we are dealing in the analysis with three different factors and three possible regulations there are 27 cases that need to be considered, some of which result in identical outputs for the three comparisons (Additional file 3).

### Clusters 4 and 7 define STATc-regulated genes

**Figure 5:**
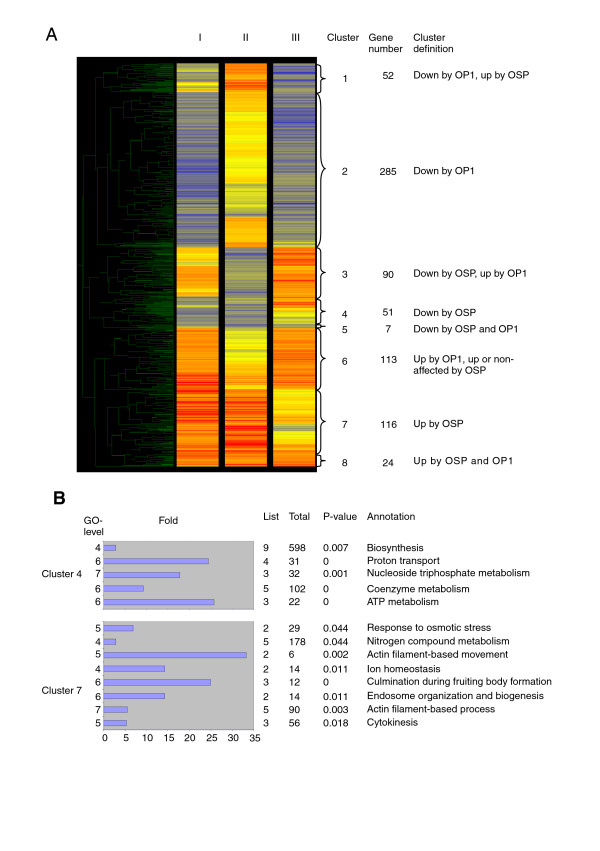
**Cluster analysis reveals clusters of STATc-regulated genes**. (A) Shared genes of Fig. 4 were clustered with GeneSpring 7.2. Eight major clusters (1–8) can be distinguished of which clusters 4 and 7 contain those genes that are solely regulated by STATc. The dendrogram is displayed on the left. The differentially regulated genes are depicted as coloured lines. The colour represents the fold of induction (red) or repression (blue) (colour scale see Fig. 3). Non-regulated genes are displayed in yellow. OP1: Osmostress induced pathway 1; OSP: Osmostress induced STATc pathway; SP: STATc pathway irrespective of osmostress. (B) GO biological process terms enriched in cluster 4 and 7. GO tree levels are shown on the left. Bar lengths represent the fold enrichment (scale x-axis). The table indicates the number of genes with a particular annotation in the cluster (List), on the entire array (Total), the significance for enrichment (P-value) and the annotation.

Of the 741 differentially regulated genes that were common between two or three of the above comparisons three genes were removed because of missing values in one of the experiments and the remaining 738 genes were subjected to cluster analysis. This way we could reduce the 27 possible cases from additional file 3 to eight major outputs or clusters (Fig. 5A). Cluster 1 houses those genes that were down-regulated by OP1 (Fig. 5A, III) but up-regulated by OSP and/or SP (Fig. 5A, II). The regulatory outcome of treated versus untreated wt cells (Fig. 5A, I) depended on the balance between OP1 and the regulation by STATc. Cluster 2 is comprised of genes that were down-regulated by OP1 and their regulation was found to be largely independent of STATc (Fig. 5A, I to III). Cluster 3 genes were up-regulated by OP1 (Fig. 5A, III) and down-regulated by STATc through OSP and/or SP (Fig. 5A, II); the OP1 response dominated the regulatory output in the experiment with treated versus untreated wt cells (Fig. 5A, I). Interestingly, the up-regulation for genes in this cluster was weaker with wt cells than with STATc knock-out cells (compare Fig. 5A, I and III). This suggests that OP1 and STATc act oppositely on these genes. Cluster 4 is characterized by genes that are down-regulated by STATc and either up-regulated or non-affected by OP1 (Fig. 5A, II and III). STATc dominates the regulatory output because the differential expression in the experiment with treated versus untreated wt cells is similar to the experiment with treated RIC versus treated STATc knock-out cells (Fig. 5A, I and II). Cluster 5 is a very small cluster with genes that are slightly down-regulated by OP1 and OSP (Fig. 5A, I to III). The last three clusters comprise genes that were up-regulated in treated versus untreated wt cells. Cluster 6 genes were up-regulated by OP1 and either unaffected or slightly up-regulated by STATc, while cluster 7 genes were up-regulated by STATc and either unaffected or slightly up-regulated by OP1 (Fig. 5A, I to III). Finally, cluster 8 genes were up-regulated by OP1 and OSP (Fig. 5A, I to III). Particularly interesting were the cluster 4 and 7 genes, which were regulated by STATc and this regulation also dominated the transciptional output of wt cells (experiment I). They constitute approximately 20% of the genes that were common between two or three experiments (see additional file 4 for a full list of these genes). To learn more about these STATc-regulated genes we subjected them to GOAT analysis. For the down-regulated genes in cluster 4 we found an enrichment of the biological process terms biosynthesis, proton transport and coenzyme metabolism, in particular ATP metabolism. The up-regulated genes in cluster 7 are characterized by an enrichment of the biological process terms response to osmotic stress, nitrogen compound metabolism, endosome organization and biogenesis, actin filament based process and culmination during fruiting body formation. The full lists of enriched biological process, molecular function and cellular component terms are available as supplementary information (Additional file 5). The results of the comparisons clearly show that STATc is the responsible regulator for a subset of the differentially regulated genes in the *Dictyostelium *osmostress response. Apparently most of the up-regulated genes in the osmotic response, which encode cytoskeletal proteins are subject to regulation by STATc. This holds also true for a subset of those genes that are involved in metabolism or the response to stress or are responsible for ion homeostasis (Fig. 5B). These results also imply that at least two signaling pathways get activated in *Dictyostelium *cells in response to hyperosmotic stress.

## Discussion

*Dictyostelium *is a powerful model system for large-scale studies of the transcriptional and translational adaptations to a changing osmotic environment. The organism is amenable to genetic manipulation, the complete genome has recently been sequenced and cDNA microarrays for global transcriptional analyses are available [34-37]. We have used these advantages of *Dictyostelium *to study its response to hyperosmotic conditions after one hour of exposure to sorbitol, in a time course experiment and by comparing the transcriptional profiles of treated or untreated wild type and STATc knock-out cells.

Treatment of *Dictyostelium *cells with 200 mM sorbitol resulted in dramatic transcriptional changes. In the time course experiment more than 800 genes were differentially regulated. A cluster analysis revealed four major clusters. Clusters 1 and 3 were characterised by up-regulated and clusters 2 and 4 by down-regulated genes (Fig. 3). The enrichment of GO terms in clusters 2 and 4 showed a down-regulation of metabolic processes (Fig. 3B, cluster 4) and of several cellular processes like chemotaxis, endocytosis and cytokinesis (Fig. 3B, cluster 2). Enrichment of genes in the latter biological processes is mainly due to the down-regulation of genes encoding cytoskeletal proteins (Fig. 3C, cluster 2). Interestingly, we also found an enrichment of genes involved in developmental processes and fruiting body formation in all four clusters of figure 3 (Fig. 3BA and additional file 2). There is a long and parallel history of the effects of osmotic pressure on vegetative cells and developing spores. The formation of dormant spores requires a high osmotic pressure exerted by the matrix between the spores, which consists largely of ammonium phosphate at a 100–200 mM concentration [38]. This leads to a raise in cAMP levels in the spore through the activation of adenylyl cyclase G (ACG) which functions as an intramolecular osmosensor [39,40]. The increase in intracellular cAMP in turn activates PKA which inhibits spore germination [41,42]. Another intriguing parallel is the tyrosine phosphorylation of actin which is induced in osmotically stimulated vegetative cells and also during sporulation [2,3,43]. Therefore, the enrichment of developmental genes is best explained if one assumes that the mechanisms which evolved to protect vegetative *Dictyostelium *cells from high osmolarity have been adapted for developmental processes. It is also noteworthy that all subunits of the vacuolar ATPase (v-ATPase) were down-regulated in a similar way (Fig. 3C, cluster 4). The v-ATPase is a rotary molecular motor that uses hydrolysis of ATP to pump protons across membranes [44]. In *Dictyostelium*, the v-ATPase is primarily localised in membranes of the contractile vacuole, an osmoregulatory organelle. Mutant *Dictyostelium *cells with reduced v-ATPase levels showed defects in endocytic function and cytosolic pH regulation but did not manifest osmoregulatory defects [45]. Our results suggest that down-regulation of the v-ATPase is part of the cellular response to hyperosmolarity that actually might increase the likelihood of cell survival. At 15 minutes post treatment only 38 genes were differentially regulated and 35 of these were up-regulated. Manual annotation revealed several interesting genes in this group (Table 2), among them STATc.

**Figure 6:**
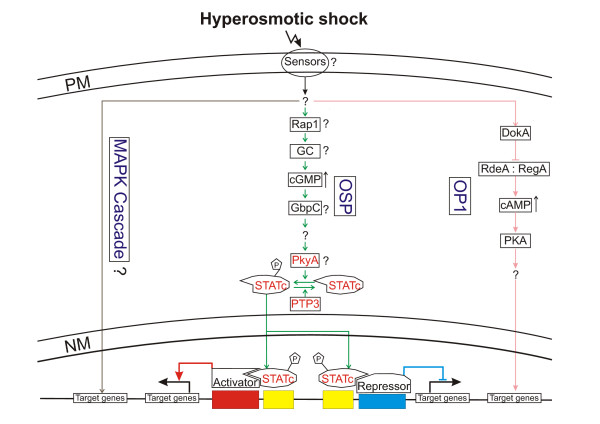
**Model**. The model depicts known and putative signal transduction pathways that might get activated in *Dictyostelium *cells in response to hypertonicity. In contrast to yeast no osmosensors are so far known in *Dictyostelium*. The OP1 (osmostress-dependent pathway 1) pathway is under control of the hybrid histidine kinase DokA and leads to elevated cAMP levels thereby activating protein kinase A (PKA). The cGMP pathway, which we named OSP (osmostress-dependent STATc pathway), leads to the activation and nuclear translocation of STATc. Other components of this pathway are probably Rap1, a guanylate cyclase (GC), GbpC, PkyA and PTP3. Based on differential regulation of putative MAPK components we propose a third signalling branch, which is under the control of a MAPK cascade, similar to yeast and mammals. Genes that were up-regulated in our experiments are in red. PM: plasma membrane; NM: nuclear membrane.

STAT proteins are latent transcription factors that dimerise upon activation through tyrosine phosphorylation followed by translocation to the nucleus where they regulate the expression of target genes [16,46]. There are four different STAT proteins encoded in the *Dictyostelium *genome, however, no STATs are present in yeast [47]. In the time course experiment with sorbitol-treated AX2 wild type cells we found STATa, b and c up-regulated, however, induction of STATc was most pronounced. Interestingly, when mammalian cells are subjected to osmotic or oxidative stress they activate JAK-STAT signalling pathways in addition to MAPK cascades [25-27]. To learn more about the role of STATc in signal transduction upon hyperosmotic shock in *Dictyostelium *we made use of a STATc null mutant [17] and compared the expression profiles of AX2 wt treated versus untreated with STATc knock-out treated versus untreated and RIC treated versus STATc knock-out treated cells. Our results show that STATc regulates the expression of approximately 20% of the more than 700 genes that were common between two or three of the above comparisons. In particular, we found that STATc dominated the osmostress-dependent expression of genes in clusters 4 and 7 (Fig. 5A). For most of these genes, including STATc itself, we observed the first transcriptional changes already 15 or 30 minutes post treatment. This period of time appears not to be sufficient for de novo expression of a STATc-dependent transcription factor that would then differentially regulate the observed target genes. Therefore, we assume that STATc directly regulates the expression of these genes. Another intriguing result was that STATc was responsible for the up- as well as down-regulation of target genes. This result can be explained if we assume that STATc acts together with a transcriptional activator for the up-regulation and/or a transcriptional repressor for the down-regulation of target genes (see Fig. 6). The nature of the putative transcriptional cofactor(s) is currently unknown. GO annotation of cluster 4 and 7 genes showed that STATc is responsible for the coordinated regulation of genes in distinct functional categories. Among the STATc-dependent genes we found an enrichment of genes involved in proton transport (due to v-ATPase subunits), actin-filament based processes, in the response to osmotic stress and culmination during fruiting body formation (Fig. 5B). During development STATc is activated by DIF, which induces the differentiation of prestalk O cells [17,48]. Previous work suggested a clear separation of the STATc target genes in development and stress [18]. In contrast, our GO results (Figs. 3B and 5B) show the enrichment of genes in the categories "development" and "culmination during fruiting body formation".

In yeast the HOG signalling pathway is responsible for the adaptation of the cells to high osmolarity. It can be activated by either of two upstream pathways, the SHO1 and the SLN1 pathway, which converge on Pbs2, a MAPKK and scaffolding protein that brings together the other components of the MAPK cascade [24]. SHO1 and SLN1 are putative yeast osmosensors and there is possibly a third one, Msb2 [49-51]. Microarray analysis showed that Msb2 and SHO1 function in parallel and regulate identical gene sets in *hog*1 mutants [51]. Investigation of the yeast transcriptional response at different osmolarities showed that different response pathways are triggered. The environmental stress response pathway is preferentially used during extreme osmotic stress, the SLN1 branch but not the Sho1 branch of the HOG pathway is used during modest osmotic stress while all three pathways contribute significantly to differential gene expression at intermediate osmolarities [52,53]. Our results of the osmostress-dependent transcriptional regulation of STATc knock-out and wt cells are best explained if one assumes two or even three signalling pathways that get activated upon subjecting *Dictyostelium *cells to hyperosmotic conditions. This conclusion is also supported by previous findings, which pointed to the activation of two independent signalling branches in the *Dictyostelium *osmostress response. The hybrid histidine kinase DokA branch and downstream effectors and the cGMP branch, that might be under the control of Rap1 [4,7,12,13]. STATc is either part of the cGMP branch or could define a third independent signalling branch. The activation and nuclear translocation of STATc upon addition of 8Br-cGMP argues for STATc being a component of the cGMP branch, however, osmotic stress induced STATc phosphorylation was still observed in a double mutant which lacked both known *Dictyostelium *guanylate cyclases [18]. Putative regulators of STATc are protein tyrosine phophatase 3 (PTP3) and PkyA, a tyrosine kinase-like protein kinase with homology to the mammalian JAK kinase (JG Williams, pers. comm.) [54,55]. While none of the known components of the DokA pathway were differentially regulated in response to hypertonicity, we find that PTP3 and PkyA, like STATc, were up-regulated. Furthermore, we found in our list of differentially regulated genes several up-regulated protein kinases that could be part of a MAPK cascade, thus raising the possibility that *Dictyostelium*, like yeast and mammals, also uses a MAPK cascade in response to osmotic stress. Fig. 6 depicts known and putative components of the *Dictyostelium *osmotic response under the assumption of three parallel signalling pathways.

A comprehensive view on the osmotic stress response requires a detailed understanding of various cellular aspects such as signal sensing and transduction, control of transport processes and metabolism and differential regulation of transcription and translation. Future work should clarify the exact role of STATc and unravel further critical components of the signal chains that get activated in *Dictyostelium *under adverse osmotic conditions.

## Conclusion

Our results demonstrate the complex response of *Dictyostelium *cells to hyperosmotic conditions. In particular massive transcriptional changes were induced that apparently lead to a down-regulation of the metabolic machinery, changes in the actin cytoskeleton and the up-regulation of the stress response. The enrichment of differentially regulated genes involved in fruiting body formation supports the assumption that the mechanisms, which evolved to protect vegetative amoebae from a hypertonic environment have been adapted for developmental processes, in particular sporulation. A key player, responsible for the expression of a subset of the differentially regulated genes, turned out to be STATc. The microarray results with STATc knock-out cells imply that at least two signalling pathways get activated in *Dictyostelium *cells subjected to hypertonicity. The OSP pathway leads to the activation and nuclear translocation of STATc followed by differential regulation of target genes. The OP1 pathway of which some components have been described previously might be under the control of the hybrid histidine kinase DokA and downstream effectors. Differential regulation of several protein kinases that could be part of a MAPK cascade indicate a possible third signalling pathway that so far remained unnoticed (Fig. 6). Unravelling the components of the signal transduction pathways involved in the response of *Dictyostelium *cells to hypertonicity will be a major challenge in the future.

## Methods

### Growth of *Dictyostelium discoideum*

The procedure was adopted from Claviez et al. [56]. *D. discoideum *AX2 and the derived transformants were grown in liquid AX2 medium containing dihydrostreptomycinsulfate (40 μg/ml) and other appropriate selective antibiotics (depending on the mutant) at 21°C either in a shaking suspension in Erlenmeyer flasks with shaking at 160 rpm or the cells were grown on petri dishes. For cell biological work, cultures were harvested at a density of 3–4 × 10^6 ^cells/ml.

### Hyperosmotic shock and determination of cell survival and cell volume

*Dictyostelium *cells were grown to a density of 3–4 × 10^6 ^cells/ml in Erlenmeyer flasks. 2 M sorbitol was added to the culture for a final concentration of 200 mM sorbitol. Samples were collected after treatment for 60 min or, for the time course experiments, samples were collected at 0, 15, 30, 45, 60, 90, and 120 min. To measure cell survival, a serial dilution was performed and approximately 100 *Dictyostelium *cells were plated onto SM agar plates overlaid with *Klebsiella aerogenes*. *Dictyostelium *plaques were counted after 2–3 days of incubation at 21°C. For cell volume determination, *Dictyostelium *cells were treated with 0, 50, 100, 200 or 400 mM sorbitol for 5 minutes. Then cells were transferred to a 100 μl microcapillary tube (BLAUBRAND^® ^intraMARK, Germany) and centrifuged at 500×g for 1 minute. The height of the cell pellet in the microcapillary was taken as a measure for cell volume.

### Confocal microscopy

*Dictyostelium *cells were harvested and resuspended in Soerensen buffer [57]. After starvation for 4 hours cells were treated with sorbitol (0, 50, 100, 200 and 400 mM) for 5 min, fixed with ice cold methanol, and then stained with a monoclonal antibody specific for actin [58] followed by anti-mouse IgG antibody conjugated with Cy5. Confocal images of immunolabelled specimens were obtained with a confocal laser scanning microscope (Leica DM/IRBE).

### RNA isolation, Northern transfer and quantitative real time PCR

*Dictyostelium *cells, treated with sorbitol or untreated, were harvested and washed twice with Soerensen buffer. RNA isolation, Northern transfer and real time PCR was essentially done as described [59].

### Microarray production, expression profiling and data analysis

We employed cDNA microarrays that carry a non-redundant set of 5,423 EST clones that were selected as part of the *Dictyostelium *cDNA project [60]. In addition, appropriate positive and negative controls as well as partial sequences of 450 selected genes were present on the array [59]. All probes were spotted in duplicate. A complete description of the microarray dataset is available at the Gene Expression Omnibus (GEO; accession number GPL1972) [61]. Microarray production, expression profiling and data analysis have been performed essentially as described [59]. Briefly, for the time course experiments we analysed six microarrays from each time point with labelled cDNAs that were derived from RNAs from three independent cultures. Dye swaps were used for the labelling of the RNA from each independent isolation. For the comparisons after 1 h of treatment with sorbitol, RNA from 8 (wt treated versus untreated) or, respectively, 3 (STATc ko treated versus untreated and RIC treated versus STATc ko treated) independent cultures was isolated, reverse transcribed, labelled and used for the hybridisation of 16 (wt treated versus untreated) or, respectively, 6 (all other comparisons) slides. Scanning was performed with the ScanArray^® ^4000 XL confocal laser scanner, signals were quantified with ScanArray Express 3.0 (PerkinElmer Life Sciences, Wellesley, USA) and Fluorescence ratios were normalised by LOWESS-normalisation using R 1.6.2 [62].

Differentially expressed genes were identified with the program Significance Analysis of Microarrays (SAM) [63]. SAM calculates a score for every gene with a t-statistic, modified for the use on microarray data. The higher the score the more reliable is the differential expression of the reported gene. This statistic is superior to a fold change cut-off or a t-test. Differentially regulated genes that were common between the different experiments were detected with the program "compare" [64]. Cluster analysis was performed with GeneSpring 7.2 [65].

GO term enrichment was analysed with GOAT [32]. A complete list of all *Dictyostelium *proteins with GO annotations is available from the GO website [30]. To identify enriched GO terms we selected those genes of the array (reference list) and of the identified clusters (gene lists) in Fig. 3A or 5A, respectively, whose gene products have GO annotations. Given a gene and a reference list, the GOAT program calculates the enrichment and statistical significance of every GO term by comparing the observed number of genes in a specific category with the number of genes that might appear in the same category if a selection performed from the same reference list were completely random.

## Abbreviations

DIF, differentiation inducing factor; GO, gene ontology; GOAT, gene ontology analysis tool; HOG, high osmolarity glycerol; MAPK, mitogen-activated protein kinase; SAM, significance analysis of microarrays; STAT, signal transducer and activator of transcription.

## Authors' contributions

JN carried out the immunofluorescence, cell biological and microarray studies. He also performed the data analysis. BT participated in the data analysis. LE conceived of the study, participated in its design and coordination and drafted the manuscript. All authors read and approved the final manuscript.

## Supplementary Material

Additional file 1 **Differentially regulated genes of the time course of sorbitol treatment**. List of all 809 genes that were differentially regulated in the time course of sorbitol treatment. The table provides the ID of the EST (expressed sequence tag), the dictyBase number (DDB ID) and the fold change at the different time points (0, 15, 30, 45, 60, 90 and 120 minutes). Click here for file

Additional file 2 **Enriched GO terms of the time course of sorbitol treatment**. Complete list of enriched biological process, molecular function and cellular component GO terms of clusters 1 to 4 of the 2 hours time course of sorbitol treatment. Click here for file

Additional file 3 **Possible regulations of target genes by different signalling pathways**. Assuming two independent signalling pathways that are activated in response to osmotic stress and a STATc pathway independent of osmostress, the table lists the possible regulatory combinations, the pathways involved in the comparisons and the expected regulatory output of target genes. Click here for file

Additional file 4 **STATc-regulated genes**. Complete list of STATc-regulated genes (clusters 4 and 7 of Fig. 5A). The table lists the dictyBase ID, the gene name, the annotation and the differential expression in the three comparisons. Click here for file

Additional file 5 **Enriched GO terms of STATc-regulated genes**. Complete list of enriched biological process, molecular function and cellular component GO terms of STATc-regulated genes in response to hyperosmolarity. Click here for file

## Figures and Tables

**Table 1:** Number of differentially expressed *Dictyostelium *genes during the two hour time course of sorbitol treatment.

**Time [min]**	**Differentially expressed genes**		
	
	**Upregulated**	**Downregulated**	**Sum**
**0**	0	0	0
**15**	35	3	38
**30**	219	266	485
**45**	244	344	588
**60**	232	351	583
**90**	120	203	323
**120**	73	138	211

**Table 2:** Selection of early differentially expressed genes with unambiguous annotation.

**DDB ID**	**Annotation**	**Differential regulation**						
		
		**T0**	**T15**	**T30**	**T45**	**T60**	**T90**	**T120**
**DDB0191392**	*sig*G: *srf*A induced gene G	1.048	2.403	4.874	6.680	5.577	1.910	0.906
**DDB0191111**	*sig*J: *srf*A induced gene J	0.989	2.289	5.638	6.472	6.361	5.625	5.244
**DDB0218006**	Homologue of human cyclin fold protein 1	0.996	2.043	3.396	2.572	1.994	0.932	0.823
**DDB0201666**	AbcB1: ABC transporter B family protein	1.095	1.981	2.916	3.316	2.797	1.627	1.049
**DDB0229894**	FcpA: putative CTD (C-terminal domain) phosphatase	1.001	1.847	2.979	3.172	3.282	2.308	1.450
**DDB0189650**	Na^+^/K^+ ^ATPase; Na^+^/K^+^-transporting ATPase alpha chain 2	1.029	1.709	3.244	3.091	3.150	1.361	0.873
**DDB0206314**	Putative transmembrane protein; 6-TM domains	1.001	1.672	2.551	2.632	2.710	1.918	1.188
**DDB0169197**	Protein contains Cyclin_N domain	1.034	1.654	2.165	1.367	1.253	0.976	1.009
**DDB0191293**	GapA: RasGTPase-activating protein	0.909	1.581	2.310	1.744	1.437	0.756	0.653
**DDB0215378**	STATc: STAT family protein	0.980	1.569	3.046	3.181	3.406	2.134	1.108
*DDB0216584*	Eucaryotic translation initiation factor 4E	0.997	0.661	0.413	0.492	0.578	0.743	0.796
*DDB0230034*	RabR: rab GTPase R	1.015	0.497	0.644	0.498	0.579	0.481	0.493
*DDB0168979*	Member of the Major Facilitator Superfamily (MFS)	0.958	0.430	0.312	0.271	0.406	0.257	0.275

SrfA: Serum Response Factor A; ABC transporter: ATP-Binding Cassette transporter; FcpA: Transcription factor II F (TFIIF)-associating CTD phosphatase; bold: up-regulated; italic: down-regulated.

**Table 3:** Osmotic stress experiments with wt, STATc ko and RIC cells.

**Comparison**	**Experiment**	**Control**	**Independent cultures**	**Microarrays**
I	wt **+**	wt **-**	8	16
II	RIC **+**	STATc ko **+**	3	6
III	STATc ko **+**	STATc ko **-**	3	6

wt: AX2 wild type cells; ko: knock-out; RIC: random integrant cells; **+**: treated; **-**: untreated.
