# Supplementary material for: STATc is a key regulator of the transcriptional response to hyperosmotic shock
Source: BMC Genomics. 2007 May 21;8:123. doi: 10.1186/1471-2164-8-123 (PMC1888708; doi:10.1186/1471-2164-8-123)
Supplement: Additional file 3 — Possible regulations of target genes by different signalling pathways. Assuming two independent signalling pathways that are activated in response to osmotic stress and a STATc pathway independent of osmostress, the table lists the possible regulatory combinations, the pathways involved in the comparisons and the expected regulatory output of target genes. [file 1471-2164-8-123-S3.doc]

## Additional file 3. Possible regulations of target genes by different signalling pathways, pathways involved in the comparisons and expected regulatory output of target genes.

| Case | Possible regulatory combinations | | | Comparison | | | Expected regulatory output | | |
| --- | --- | --- | --- | --- | --- | --- | --- | --- | --- |
|  | OP1 | OSP | SP | WT +/WT - | RIC +/STATc ko + | STATc ko +/STATc ko - | WT +/WT - | RIC +/  STATc ko + | STATc ko +/  STATc ko - |
|  |  |  |  | Pathways involved | | |  |  |  |
|  |  |  |  | OP1*OSP*SP/SP | OP1*OSP*SP/OP1 | OP1 | Expression | | |
| 1 | ↓ | ↓ | ↓ | OP1*OSP | *OSP***SP* | *OP1* | ↓ | ↓ | ↓ |
| 2 | ↑ | ↓ | ↓ | **OP1****OSP* | *OSP***SP* | OP1 | ↑/↓/0 | ↓ | ↑ |
| 3 | 0 | ↓ | ↓ | OP1**OSP* | *OSP***SP* | OP1 | ↓ | ↓ | 0 |
| 4 | ↓ | ↓ | ↑ | *OP1***OSP* | *OSP****SP** | *OP1* | ↓ | ↑/↓/0 | ↓ |
| 5 | ↑ | ↓ | ↑ | **OP1****OSP* | *OSP****SP** | OP1 | ↑/↓/0 | ↑/↓/0 | ↑ |
| 6 | 0 | ↓ | ↑ | OP1**OSP* | *OSP****SP** | OP1 | ↓ | ↑/↓/0 | 0 |
| 7 | ↓ | ↓ | 0 | *OP1***OSP* | *OSP**SP | *OP1* | ↓ | ↓ | ↓ |
| 8 | ↑ | ↓ | 0 | **OP1****OSP* | *OSP**SP | OP1 | ↑/↓/0 | ↓ | ↑ |
| 9 | 0 | ↓ | 0 | OP1**OSP* | *OSP**SP | OP1 | ↓ | ↓ | 0 |
| 10 | ↓ | ↑ | ↓ | *OP1****OSP** | **OSP****SP* | *OP1* | ↑/↓/0 | ↑/↓/0 | ↓ |
| 11 | ↑ | ↑ | ↓ | **OP1*****OSP** | **OSP****SP* | OP1 | ↑ | ↑/↓/0 | ↑ |
| 12 | 0 | ↑ | ↓ | OP1***OSP** | **OSP****SP* | OP1 | ↑ | ↑/↓/0 | 0 |
| 13 | ↓ | ↑ | ↑ | *OP1****OSP** | **OSP*****SP** | *OP1* | ↑/↓/0 | ↑ | ↓ |
| 14 | ↑ | ↑ | ↑ | **OP1*****OSP** | **OSP*****SP** | OP1 | ↑ | ↑ | ↑ |
| 15 | 0 | ↑ | ↑ | OP1***OSP** | **OSP*****SP** | OP1 | ↑ | ↑ | 0 |
| 16 | ↓ | ↑ | 0 | *OP1****OSP** | **OSP***SP | *OP1* | ↑/↓/0 | ↑ | ↓ |
| 17 | ↑ | ↑ | 0 | **OP1*****OSP** | **OSP***SP | OP1 | ↑ | ↑ | ↑ |
| 18 | 0 | ↑ | 0 | OP1***OSP** | **OSP***SP | OP1 | ↑ | ↑ | 0 |
| 19 | ↓ | 0 | ↓ | *OP1**OSP | OSP**SP* | *OP1* | ↓ | ↓ | ↓ |
| 20 | ↑ | 0 | ↓ | **OP1***OSP | OSP**SP* | OP1 | ↑ | ↓ | ↑ |
| 21 | 0 | 0 | ↓ | OP1*OSP | OSP**SP* | OP1 | 0 | ↓ | 0 |
| 22 | ↓ | 0 | ↑ | *OP1**OSP | OSP***SP** | *OP1* | ↓ | ↑ | ↓ |
| 23 | ↑ | 0 | ↑ | **OP1***OSP | OSP***SP** | OP1 | ↑ | ↑ | ↑ |
| 24 | 0 | 0 | ↑ | OP1*OSP | OSP***SP** | OP1 | 0 | ↑ | 0 |
| 25 | ↓ | 0 | 0 | *OP1**OSP | OSP*SP | *OP1* | ↓ | 0 | ↓ |
| 26 | ↑ | 0 | 0 | **OP1***OSP | OSP*SP | OP1 | ↑ | 0 | ↑ |
| 27 | 0 | 0 | 0 | OP1*OSP | OSP*SP | OP1 | 0 | 0 | 0 |

OP1: Osmostress induced pathway 1; OSP: Osmostress induced STATc pathway; SP: STATc pathway irrespective of osmostress; wt: AX2 wild type cells; ko: knock-out; RIC: random integrant cells; ↑ or bold: up-regulated; ↓ or italic: down-regulated; 0 or underlined: non-regulated; +: treated; -: untreated
